# Asymptomatic erythematous plaques on the trunk

**DOI:** 10.1016/j.jdcr.2024.01.018

**Published:** 2024-01-26

**Authors:** Madison Novice, Carrie L. Vuong, Stephanie S. Lee, Brian Hinds, Bonita Kozma

**Affiliations:** a University of Michigan Medical School, Ann Arbor, Michigan; b Department of Dermatology, University of California San Diego, San Diego, California; c Department of Dermatology, University of Michigan, Ann Arbor, Michigan

**Keywords:** cutaneous oncology, Waldenstrom macroglobulinemia

## History

**Fig 1:**
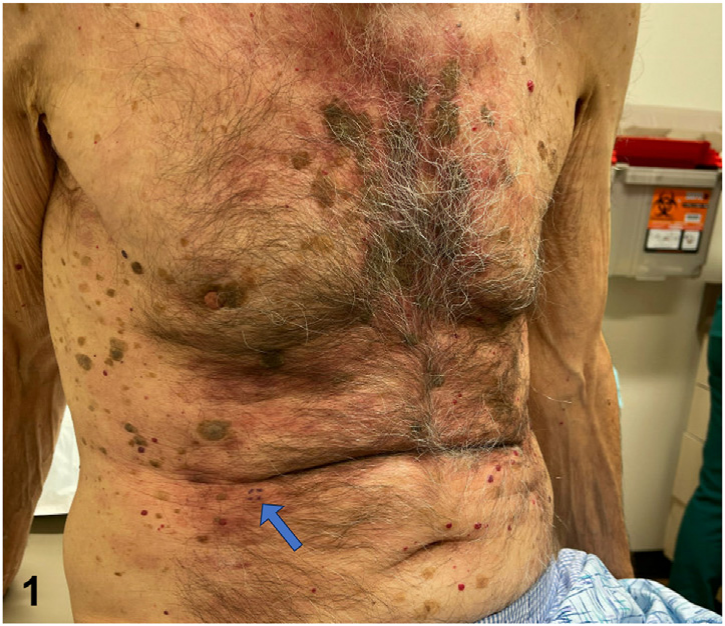

**Fig 2:**
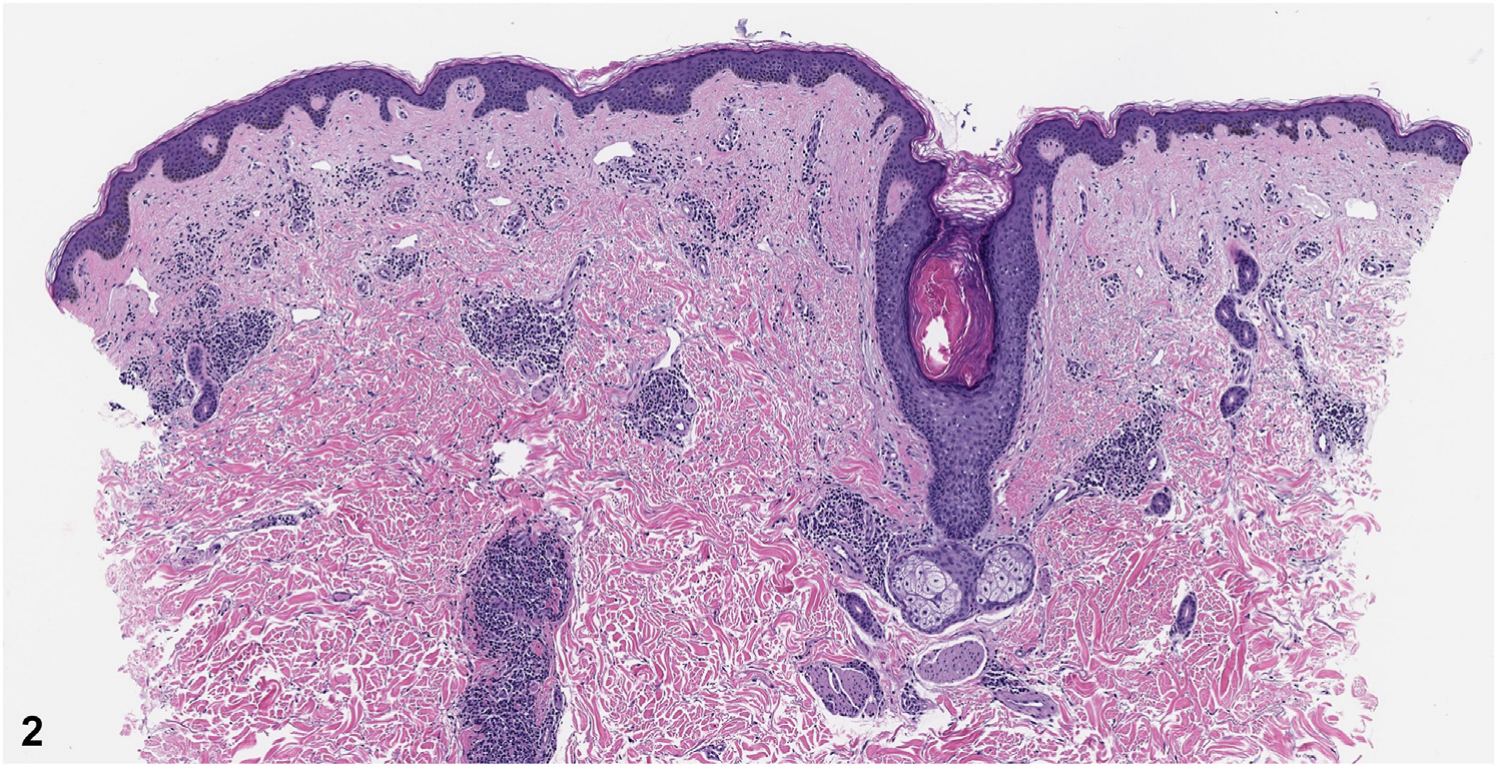

**Fig 3:**
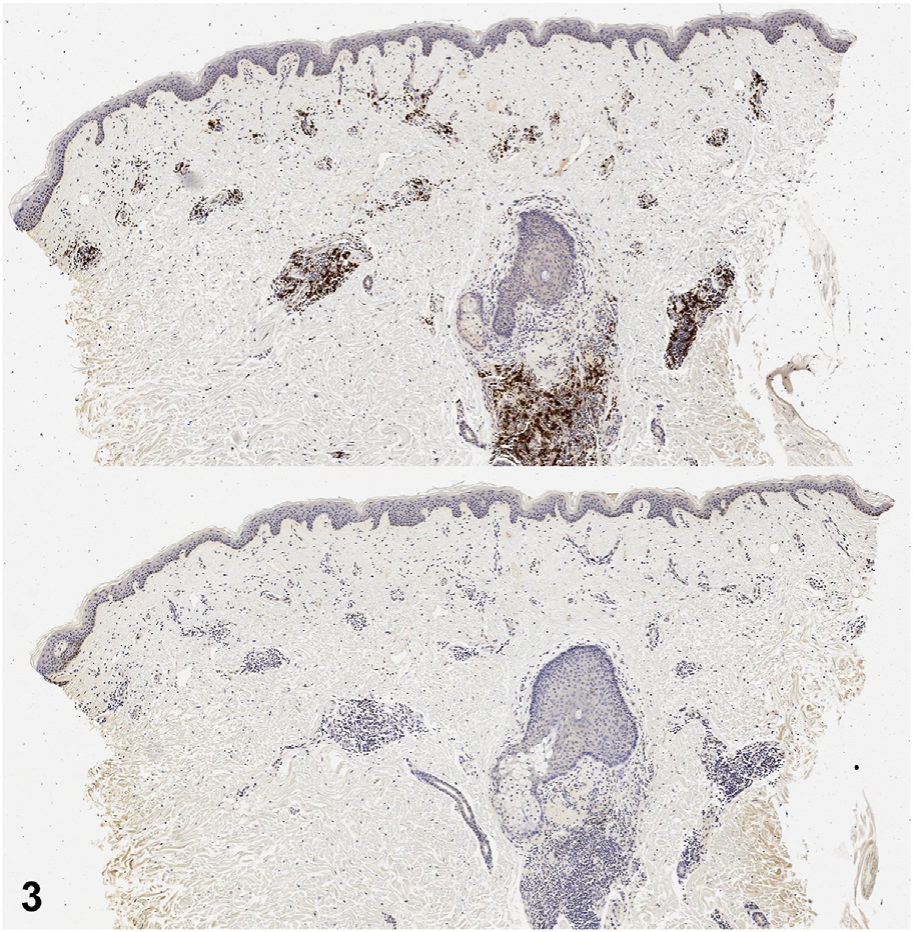

An 81-year-old male with a history of nonmelanoma skin cancer and Waldenstrom macroglobulinemia (WM) presented for evaluation of an asymptomatic rash on his trunk present for years with minimal change. He had no history of radiation treatment. Review of systems was negative for fever, unintentional weight loss, night sweats, confusion, and double vision. On physical examination, he had violaceous to erythematous plaques on the anterior trunk and lower back (Fig 1). A 4-mm punch biopsy demonstrated a patchy perivascular and periadnexal lymphoplasmacytic infiltrate (Fig 2). CD117 (mast cell marker) and CD123 (plasmacytoid dendritic cell marker) stains were negative. Chromogenic in situ hybridization revealed predominately kappa-restricted (clonal) plasma cells (Fig 3).

**Question 1: What is the most likely diagnosis?**

A. Mycosis fungoides
B. Tumid lupus erythematosus
C. Cutaneous WM
D. Primary cutaneous marginal zone lymphoma
E. Multiple myeloma

**Answers:**

A. Mycosis fungoides – Incorrect. While patch/plaque stage mycosis fungoides was originally favored clinically, expected histopathologic features include epidermotropism of atypical lymphocytes, superficial lichenoid infiltrate of lymphocytes and histiocytes, and/or intraepidermal collections of atypical lymphocytes (Pautrier microabscesses) were not seen.
B. Tumid lupus erythematosus – Incorrect. Tumid lupus erythematosus can have violaceous plaques without surface change; however, the lesions are most commonly photo-distributed. Histopathologic examination demonstrates increased dermal mucin and a superficial and deep predominately lymphocytic infiltrate with perieccrine extension.
C. Cutaneous WM – Correct. Our patient’s history and biopsy findings are most consistent with cutaneous WM. Cutaneous findings are seen in <5% of cases and divided into the following 2 categories: (1) specific manifestations secondary to direct neoplastic involvement of the skin and (2) nonspecific manifestations secondary to hyperviscosity and cryoglobulinemia. Infiltration of clonal lymphoplasmacytic cells in the skin, as seen on our patient’s biopsy, results in erythematous plaques of variable size, most often on the face/trunk.^1^^,^^2^
D. Primary cutaneous marginal zone lymphoma – Incorrect. While primary cutaneous marginal zone lymphoma can have a similar clinical presentation, histopathology would demonstrate dermal infiltrates of mature B-cells/plasma cells which can be nodular or diffuse with varying degrees of plasmacytoid differentiation (CD123 expected to be positive). There are also usually lymphoid follicles with reactive germinal centers.
E. Multiple Myeloma – Incorrect. Plasmacytomas in multiple myeloma can have erythematous to violaceous plaques or nodules due to direct infiltration of skin, occasionally emanating from underlying bony foci. Histopathology demonstrates deep dermal and subcutaneous plasma cell infiltrates in sheets that are positive for CD38 and CD138 and negative for B-cell markers.^1^

**Question 2: Which of the following targeted therapies have demonstrated long-term safety and efficacy in the management of symptomatic WM?**

A. Vemurafenib
B. Bevacizumab
C. Imatinib
D. Zanubrutinib
E. Trastuzumab

**Answers:**

A. Vemurafenib – Incorrect. Vemurafenib is a V-raf Murine Sarcoma Viral Oncogene Homolog B inhibitor approved for the treatment of metastatic melanoma with a V600 mutation and Erdheim-Chester disease (non-Langerhans histiocytic disorder).
B. Bevacizumab – Incorrect. Bevacizumab is a vascular endothelial growth factor inhibitor approved as first-line therapy in the management of nonsmall cell lung cancer and metastatic colorectal cancer.
C. Imatinib – Incorrect. Imatinib is a BRC-ABL tyrosine kinase inhibitor approved for the treatment of chronic myeloid leukemia. Imatinib is also used in the management of unresectable, recurrent, or metastatic dermatofibrosarcoma protuberans and some cases of systemic mastocytosis.
D. Zanubrutinib – Correct. Zanubrutinib is a selective next-generation Bruton tyrosine kinase inhibitor. Long-term follow-up from a randomized phase III trial demonstrated zanubrutinib’s efficacy and safety in managing WM.^3^ Our patient was treated with zanubrutinib, which led to significant improvement in cutaneous lesions.
E. Trastuzumab – Incorrect. Trastuzumab is a human epidermal growth factor receptor 2 inhibitor used for the management of human epidermal growth factor receptor 2–positive breast cancer or human epidermal growth factor receptor 2–overexpressing metastatic gastric or gastroesophageal junction adenocarcinoma.

**Question 3: Which gene mutation detected on skin biopsy can help diagnose WM?**

A. MYD88 L265P
B. BRCA1-associated protein 1
C. Protein patched homolog 1
D. V-raf Murine Sarcoma Viral Oncogene Homolog B V600 E
E. Tumor protein 53

**Answers:**

A. MYD88 L265P – Correct. The MYD88 L265P mutation is present in >90% of cases of WM, which can be useful in differentiating WM from diseases that share similar clinical and immunohistochemical features, such as marginal zone lymphoma. In patients with low tumor burden in the bone marrow, polymerase chain reaction on skin biopsy can have higher sensitivity in detecting this mutation.^4^
B. BRCA1-associated protein 1 – Incorrect. Inherited variants of this mutation are associated with a tumor predisposition syndrome. There is increased risk of uveal and cutaneous melanoma, atypical spitz nevi, renal cell carcinoma, mesothelioma, melanocytic BRCA1-associated protein 1 mutated atypical intradermal tumors, and basal cell carcinoma.^5^
C. Protein patched homolog 1 – Incorrect. This mutation is associated with basal cell nevus syndrome (inherited) and somatic mutations are associated with sporadic basal cell carcinoma.
D. V-raf Murine Sarcoma Viral Oncogene Homolog B V600 E – Incorrect. This mutation can be found in melanoma, Langerhans cell histiocytosis, and some types of non-Hodgkin lymphoma.
E. Tumor protein 53 – Incorrect. Tumor protein 53 mutations are associated with various malignancies, including some types of lymphoma, but they are neither specific to WM nor typically detected on skin biopsy in this disease.

## Conflicts of interest

None disclosed.
